# Insights into CLC-0’s Slow-Gating from Intracellular Proton Inhibition

**DOI:** 10.3390/ijms25147796

**Published:** 2024-07-16

**Authors:** Hwoi Chan Kwon, Robert H. Fairclough, Tsung-Yu Chen

**Affiliations:** 1 Biophysics Graduate Program, University of California, Davis, CA 95618, USA; hwoichankwon@gmail.com (H.C.K.); rhfairclough@ucdavis.edu (R.H.F.); 2 Department of Neurology, University of California, Davis, CA 95618, USA; 3 Center for Neuroscience, University of California, 1544 Newton Court, Davis, CA 95618, USA

**Keywords:** CLC channel, slow gating, CLC-0, proton inhibition, inactivation

## Abstract

The opening of the *Torpedo* CLC-0 chloride (Cl^−^) channel is known to be regulated by two gating mechanisms: fast gating and slow (common) gating. The structural basis underlying the fast-gating mechanism is better understood than that of the slow-gating mechanism, which is still largely a mystery. Our previous study on the intracellular proton (H^+^_i_)-induced inhibition of the CLC-0 anionic current led to the conclusion that the inhibition results from the slow-gate closure (also called inactivation). The conclusion was made based on substantial evidence such as a large temperature dependence of the H^+^_i_ inhibition similar to that of the channel inactivation, a resistance to the H^+^_i_ inhibition in the inactivation-suppressed C212S mutant, and a similar voltage dependence between the current recovery from the H^+^_i_ inhibition and the recovery from the channel inactivation. In this work, we further examine the mechanism of the H^+^_i_ inhibition of wild-type CLC-0 and several mutants. We observe that an anion efflux through the pore of CLC-0 accelerates the recovery from the H^+^_i_-induced inhibition, a process corresponding to the slow-gate opening. Furthermore, various inactivation-suppressed mutants exhibit different current recovery kinetics, suggesting the existence of multiple inactivated states (namely, slow-gate closed states). We speculate that protonation of the pore of CLC-0 increases the binding affinity of permeant anions in the pore, thereby generating a pore blockage of ion flow as the first step of inactivation. Subsequent complex protein conformational changes further transition the CLC-0 channel to deeper inactivated states.

## 1. Introduction

The CLC family includes transmembrane proteins of two functional categories: anion conducting channels and Cl^−^/H^+^ antiporters [1,2]. The anion channels include CLC-0, CLC-1, CLC-2, CLC-Ka, and CLC-Kb, while the Cl^−^/H^+^ antiporters are CLC-3, CLC-4, CLC-5, CLC-6, and CLC-7 [3,4,5,6,7,8,9,10,11]. All CLC family members consist of two identical subunits, with each of the two subunits forming an independent anion transport protopore pathway across the lipid bilayer [12,13,14,15,16]. CLC-0 from the electric organ of the *Torpedo* fish [11] is regarded as the prototype channel of the family and has been extensively studied in structure-function relations. Based on the single-channel electrophysiological study of CLC-0, it is known that two kinetically distinct gating mechanisms exist in this channel, the fast- and the slow-gating mechanisms [17,18]. 

**Figure 1:**
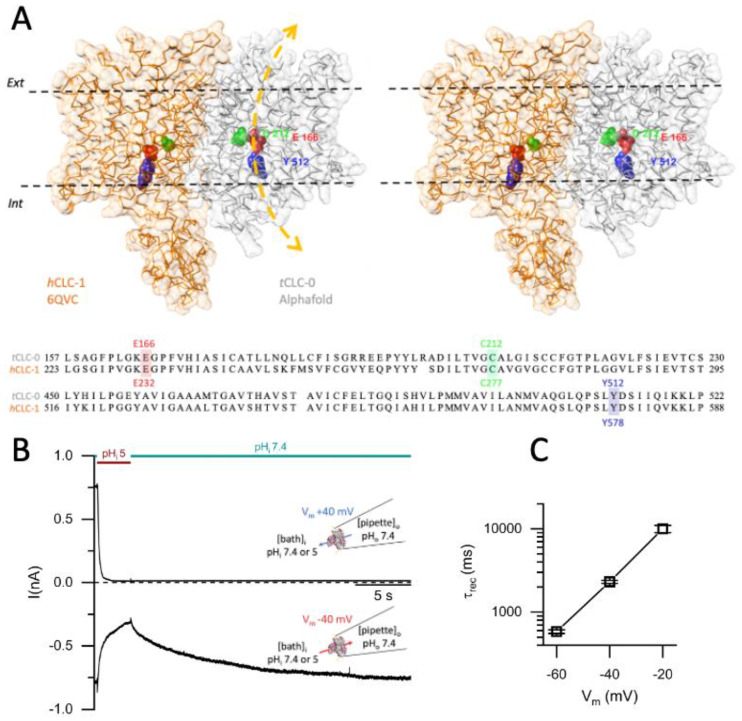
The structure of CLC channels and inhibition of WT CLC-0 by acidic pH_i_ (**A**) Cross-eyed stereo views of the CLC heterodimer constructed from hCLC-1 (**left**, colored in orange) and CLC-0 (**right**, colored in grey). See Section 4. Both the hCLC-1 and CLC-0 structures are presented as a wire frame of the backbone. The transparent surface of the proteins is also depicted. The black-dotted lines indicate the location of the lipid bilayer headgroups. The space-filled residues, glutamate (red, E166 of CLC-0 & E232 of CLC-1), cysteine (green, C212 of CLC-0 & C277 of CLC-1), and tyrosine (blue, Y512 of CLC-0 & Y578 of CLC-1) are examined in this study. The yellow-dotted arrow shown in the CLC-0 monomer indicates the Cl^−^ permeation pathway. In the CLC-0 structure, Helix A and the C-terminal domain are removed for presentation clarity. The sequence alignment of CLC-0 and CLC-1 is shown below using the same color to highlight the space-filled residues in the structures. (**B**) Th time course of the acid-induced inhibition and current recovery of WT CLC-0 at ±40 mV in 140 mM Cl^−^ on both sides of the membrane. The horizontal-colored lines up top indicate the solution exchange between pH_i_ = 5 (red) and pH_i_ = 7.4 (green). Note that current recovery only occurs at −40 mV (**lower panel**) with anion direction inside → outside. Insets show the inward and outward Cl^−^ flux directions depicted with blue and red arrows, respectively. (**C**) The current recovery time constant (τ_rec_) as a function of V_m_. The value of τ_rec_ is ~600 ms, ~2000 ms, and ~10,000 ms at −60 mV, −40 mV, and −20 mV, respectively) (n = 4–6).

In single-channel recording traces of wild-type (WT) CLC-0, the fast gating occurs with bursts of channel opening and closing activities on a millisecond (ms) timescale, highlighting the independent activity of the two protopores. This gating mechanism is voltage dependent with the pore opening favored by membrane depolarization [19,20]. Structural studies indicate that the fast-gate closure likely results from the occlusion of the pore by the sidechain of a highly conserved glutamate (E_gate_), which in CLC-0 is the amino acid residue E166 (colored in red in the Figure 1A amino acid sequence) [12,13,14,21]. The opening of the fast gate thus may come from a relief of the occlusion through a competition between the E_gate_ with anions in the pore or from the protonation of the E_gate_ [22,23,24,25,26], based on the well-documented phenomena that extracellular and intracellular Cl^−^ and H^+^ increase the open probability (*P*_o_) of the fast gate [19,27,28,29].

In between the fast-gating burst activities in the single-channel recording traces, CLC-0 undergoes long periods of quiescence of activity of both pores that persists for seconds, referred to as inactivation. This has been proposed to be due to the closure of another “gate” different from the fast gate [30]. Because of the slow kinetics of this gating, which opens and closes the two protopores virtually simultaneously, this inactivation gating mechanism is also called slow gating or common gating. As distinguished from the fast gating, membrane hyperpolarization favors the slow-gate opening [31,32]. Although slow gating is less well-characterized than fast gating, it has been suggested that slow gating involves complex conformational changes in several regions of the channel protein, including the dimer interface [33,34,35], the selectivity filter [36,37], and the C-terminal domain [38]. A complex conformational change would explain the observed high temperature dependence of the slow gating (Q_10_ ~40) [32]. 

Our previous study [39] showed that lowering the intracellular pH (pH_i_) induces an inversion of the voltage dependence of the overall current activation of WT CLC-0—a phenomenon resulting from the inhibition of CLC-0 by intracellular H^+^ (H^+^_i_). As opposed to the normal voltage dependence (which is depolarization-activated), WT CLC-0 in acidic pH_i_ is activated by membrane hyperpolarization like that of the inward-current-rectifying mutant V490W [39], or that of the homologous Cl^−^ channel, CLC-2 [40,41]. This phenomenon is significant, as the inversion of the voltage dependence of the CLC channel opening has been observed in many CLC-1 mutants causing human myotonia disease [3,42]. On the other hand, CLC-2, a CLC channel expressed in the aldosterone-secreting cells in adrenal glands, is normally opened by membrane hyperpolarization, and the CLC-2 channelopathy causing an opening by more depolarized voltages leads to a high secretion of aldosterone and thus hypertension [43,44,45,46,47]. Thus, understanding the mechanism of the hyperpolarization-induced opening of CLC-0 is pharmacologically important because it could lead to a therapeutic pathway for targeting the mammalian CLC channels in treating myotonia or hyperaldosteronism. 

Several of our previous experimental observations led us to conclude that the inhibition of CLC-0 by H^+^_i_ results from a facilitation of slow-gate closure [39]. First, the voltage-dependence and the high temperature-dependence observed in the inhibition by acidic pH_i_ [39] are like those in slow gating. Secondly, titrating the H^+^_i_ inhibition rate with various pH_i_ conditions reveals that the H^+^_i_ inhibition rate extrapolated to neutral pH_i_ is like the slow-gate closing rate. Thirdly, the mutants documented to have the slow-gate closure suppressed, namely C212S and Y512A, are less sensitive to acidic pH_i_ inhibition than the WT channel. The dramatic difference in the current recovery from the H^+^_i_-induced inhibition between WT channels in a positive or a negative membrane voltage (V_m_) is noteworthy—the current can recover at −40 mV but not at +40 mV under experimental conditions with symmetrical 140 mM Cl^−^ on both sides of the membrane [39]. Interestingly, in the slow-gate closure suppressed mutants, such as C212S and Y512A [35,37], the H^+^_i_-inhibited current can recover at both hyperpolarized and depolarized V_m_s [39]. In this study, we aim to examine the difference of the H^+^_i_ inhibitions among the WT and mutant channels to gain a better understanding of the slow-gating mechanism of CLC-0.

## 2. Results

### 2.1. The Magnitude and Direction of Anion Flux Affect the Recovery of the Wild-Type CLC-0 Current from H^+^_i_-Induced Inhibition

Our previous study showed that current inhibition of CLC-0 by acidic pH_i_ reflects slow-gate closure. Thus, the current recovery from acidic pH_i_ inhibition would represent slow-gate opening. A similar voltage dependence of the slow gate opening and that of the recovery from acidic pH_i_-induced inhibition supports this argument. Examples of the dependence of the current recovery from the H^+^_i_ inhibition of WT CLC-0 on V_m_ is illustrated in Figure 1B. In symmetric 140 mM [Cl^−^], the recovery from the H^+^_i_ inhibition is virtually undetectable at +40 mV even after 30 s, while the current recovery is readily observed at −40 mV. We first examine the current recovery process at negative V_m_ with a mono-exponential equation (Equation (1) in Section 4, i = 1). The average current recovery time constants (τ_rec_s) at three different V_m_s are depicted in Figure 1C, displaying an increase in the rate of current recovery with an increase in hyperpolarization!

**Figure 2:**
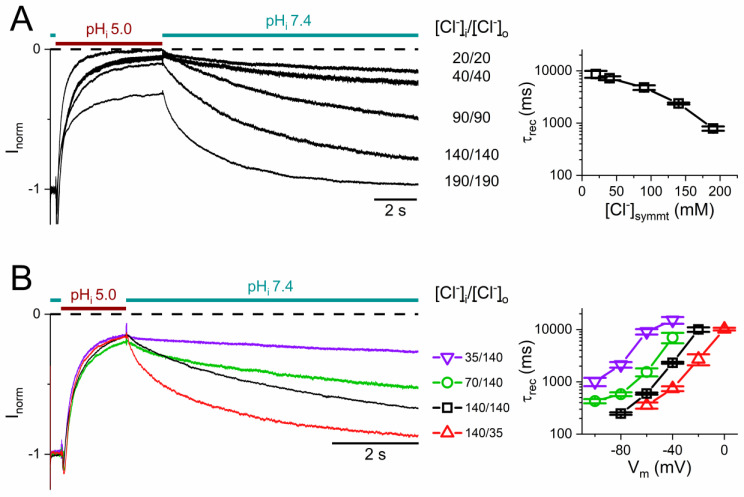
Current recovery from acid-induced inhibition under various [Cl^−^] and V_m_ conditions. The recorded current was normalized to the current immediately before the application of the pH 5 solution. The colored horizontal lines indicate the exchange of the intracellular solutions between pH_i_ 7.4 and 5.0. ((**A**), **left**) A time course of the acid-induced inhibition and current recovery of WT CLC-0 under various symmetrical [Cl^−^] (in mM) at −40 mV. ((**A**), **right**) τ_rec_ as a function of [Cl^−^] (n = 5–7). ((**B**), **left**) A time course of the acid-induced current inhibition and recovery of WT CLC-0 at −40 mV under various asymmetrical Cl^−^ conditions with a [Cl^−^]_i_/[Cl^−^]_o_ ratio ranging from (in mM) 35/140, 70/140, 140/140, to 140/35. Notice that the current recovers faster with increasing the [Cl^−^]_i_/[Cl^−^]_o_ ratio. ((**B**), **right**) τ_rec_ from analyzing the current recovery at various V_m_s in asymmetrical Cl^−^ conditions (n = 4–7).

The voltage dependence of the recovery from the H^+^_i_ inhibition agrees with the voltage dependence of the slow-gate opening, with a larger slow-gate *P*_o_ at a more negative V_m_ [17,48,49]. To further examine the mechanism underlying the current recovery from H^+^_i_ inhibition, we compared the current recovery kinetics in various symmetrical [Cl^−^] ranging from 20 to 190 mM (Figure 2A, left), which, at −40 mV, results in an increase in the recovery rate from a τ_rec_ of ~10,000 ms in 20 mM [Cl^−^] to a τ_rec_ of ~800 ms in 190 mM [Cl^−^] (Figure 2A, right), suggesting that the different rates of the current recovery are affected by the magnitude of the Cl^−^ flux through the pore because a higher [Cl^−^] generates a larger Cl^−^ flux through the channel pore. Furthermore, we suspect that the current recovery rate may also depend on the direction of the Cl^−^ flux because virtually no current recovery is observed at a positive V_m_ in symmetrical [Cl^−^] conditions (Figure 1B top panel). 

We next examined the current recovery under asymmetrical [Cl^−^] conditions, in which the Cl^−^ reversal potential (E_Cl_ ~ ln([Cl^−^]_i_/[Cl^−^]_o_)) was altered. Increasing intracellular [Cl^−^]_i_ relative to extracellular [Cl^−^]_o_ results in a positive shift of E_Cl_. The change in E_Cl_ could result in a change in the magnitude and direction of the driving force for Cl^−^ permeation (Cl^−^ driving force = V_m_ − E_Cl_). In the left panel of Figure 2B, the channel inhibition by H^+^_i_ at −40 mV was examined in four different gradients ([Cl^−^]_i_/[Cl^−^]_o,_ in mM): 35/140, 70/140, 140/140, and 140/35. Averaged results of similar experiments at various V_m_s are summarized in the right panel of Figure 2B, which shows an increase of recovery rate with an increasing [Cl^−^]_i_/[Cl^−^]_o_ ratio at any particular V_m_. The results from these V_m_ and [Cl^−^] experiments reaffirm the critical roles of both the direction and the magnitude of the Cl^−^ flux in determining the recovery rate. Together with the experimental results shown in Figure 2A, it appears that a larger Cl^−^ efflux results in a faster current recovery!

**Figure 3:**
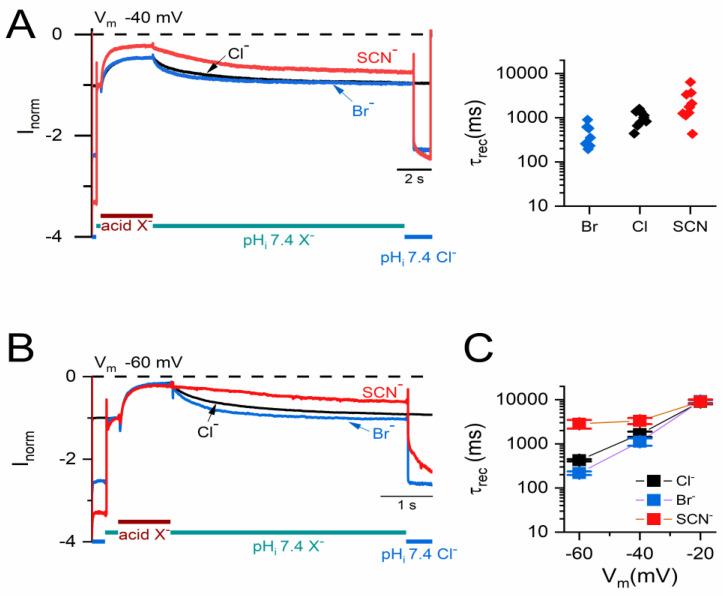
Current recovery from acid-induced inhibition of WT CLC-0 in different intracellular anions. [Cl^−^]_o_ = 140 mM. (**A**) A time course of the current inhibition and recovery at −40 mV. The intracellular solution contained 4 mM Cl^−^ and 136 mM of SCN^−^ (red), Cl^−^ (black), or Br^−^ (blue). The current was normalized to the current immediately before the application of the intracellular acidic solutions (pH_i_ 5.0). The right panel shows the τ_rec_ from fitting the kinetics of current recovery of individual recordings with a mono-exponential function. (**B**) A time course of the acid-induced inhibition and current recovery at −60 mV in different intracellular anions. The ionic conditions were like those in A, except the pH_i_ used for inhibiting the current was 4.5. (**C**) The averaged τ_rec_ at different V_m_s in different intracellular anions (n = 4–7).

To examine whether other permeant anions play a similar role as that of Cl^−^ in affecting the current recovery, the H^+^_i_ inhibition was examined under experimental conditions with 140 mM extracellular Cl^−^ (pH = 7.4) and intracellular solutions consisting of 4 mM Cl^−^ plus 136 mM various anions such as SCN^−^ (red), Cl^−^ (black), or Br^−^ (blue) (Figure 3). If the intracellular solution contains only 4 mM Cl^−^ but not other extra anions, a very slow current recovery was expected because the τ_rec_ was already longer than 10,000 ms even with 35 mM Cl^−^ (Figure 2B). As shown in Figure 3A, in which all the experiments were conducted at −40 mV, a complete recovery of the H^+^_i_-inhibited current is observed in SCN^−^ or Br^−^, with both τ_rec_s comparable to those in 140 mM [Cl^−^]_i_.

The current recovery processes in SCN^−^ or Br^−^ at −20 and −60 mV were also examined. Examples of the recording traces at −60 mV are presented in Figure 3B while the averaged τ_rec_s in Cl^−^, SCN^−^, and Br^−^ at −20, −40, and −60 mV are compared in Figure 3C. The τ_rec_s in different intracellular anions are nearly identical to each other at −20 mV while they differ at more negative V_m_s. The reversal potentials (E_rev_) measured in this study with intracellular Br^−^, Cl^−^, and SCN^−^ (all with 140 mM [Cl^−^]_o_) were −10, 0, and +7 mV, respectively, similar to those reported previously [50]. The values of E_rev_ can reflect the relative binding affinities of these anions to the pore. For example, an E_rev_ of +7 mV measured with nearly identical [SCN^−^]_i_ and [Cl^−^]_o_ would suggest SCN^−^ binds to the pore more tightly than Cl^−^, while the −10 mV E_rev_ with [Br^−^]_i_ and [Cl^−^]_o_ suggests Br^−^ binds to the pore less tightly than Cl^−^ [51]. The tighter the anion binding in the pore, the smaller the anion efflux. This might explain the slower current recovery rate in [SCN^−^] and the faster recovery rate in [Br^−^] compared to that in [Cl^−^]_i_. Furthermore, Figure 3C appears to show a larger difference in τ_rec_s among these anionic conditions as the V_m_ becomes more negative (hyperpolarized). These experiments reiterate a complex interaction between anion flux and V_m_ in the channel recovery from inactivation. 

### 2.2. Analyzing H^+^_i_ Inhibition in the Inactivation-Suppressed Mutants of CLC-0

**Figure 4:**
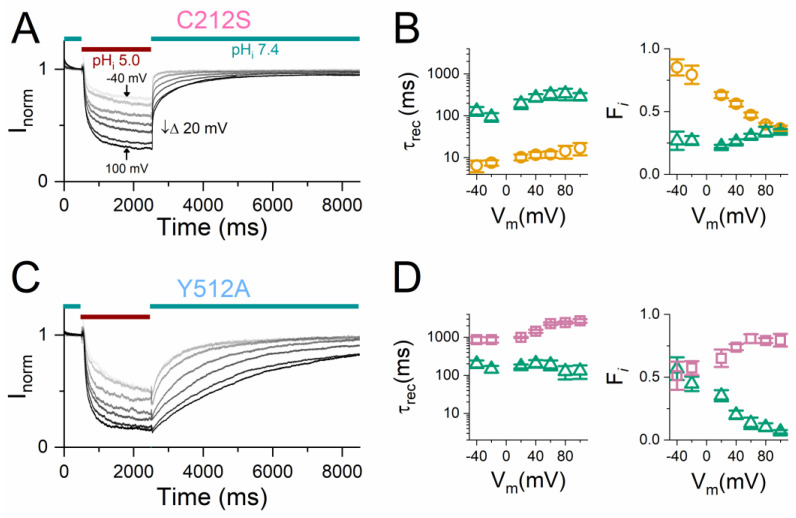
Voltage-dependent current recovery kinetics in inactivation-suppressed mutants of CLC-0. (**A**,**C**) A time course of acid-induced inhibition and current recovery of C212S (**A**), Y512A (**C**). The experiments were conducted at V_m_s from −40 mV to +100 mV in 20 mV increment except 0 mV. The red and green horizontal lines represent the patch exposure to pH_i_ = 5.0 and pH_i_ = 7.4, respectively. The recording traces in each panel are from the same patch. For comparison, the current is normalized to the current immediately before the application of pH_i_ 5.0 solution. Note that negative currents were obtained at V_m_ < 0, but the current value became positive after normalization. (**B**,**D**) The averaged τ_rec_ (**left panels**) and fractions (F*_i_*, **right panels**) of the two exponential components from fitting current recovery kinetics with bi-exponential equations. See Section 4 for the definition of F*_i_*. The data in panels (**B**) (C212S) and (**D**) (Y512A) were from experiments like those shown in panels (**A**,**C**), respectively (n = 5–6). Open gold circles, open green triangles, and open purple squares represent the ~10 ms, ~100 ms, and ~1000 ms exponential components, respectively.

The inactivation of WT CLC-0 at a positive V_m_ is difficult to study with no detectable current recovery (Figure 1B). However, the current recovery from H^+^_i_ inhibition in the C212S and Y512A mutants can be observed at positive as well as negative V_m_s [39], as illustrated in Figure 4. It appears that the current recovery process of C212S cannot be described well with a mono-exponential function (Figure 4A). We first fit the current recovery of the C212S mutant with a bi-exponential equation (see Equation (1) in Section 4), yielding a fast (τ_rec_, ~10 ms, left panel of Figure 4B, gold circles) and a slow time constant (τ_rec_ ~100 ms, left panel of Figure 4B, green triangles). It should be emphasized that although the fast component has a time constant of ~10 ms, this fast recovery component is associated with slow-gate opening but not with fast-gate opening because an increase of pH_i_ reduces the fast-gate *P*_o_ of CLC-0. The curve fitting also generates amplitudes of the corresponding exponential components (A*_i_*), which reflect the fraction of each component in the total recovered current (F*_i_* in Equation (2)). The two τ_rec_s (left panel of Figure 4B) are not voltage dependent, but the fraction of the two components from the exponential fit are (right panel of Figure 4B). The depolarization of V_m_ leads to a decrease in the fraction of the fast component (right panel of Figure 4B, gold circles), while the fraction of the slow component increases (right panel of Figure 4B, green triangles). Like C212S, Y512A exhibits complex current recovery kinetics requiring a bi-exponential fit (Figure 4C). However, the two τ_rec_s of the exponential components (fast τ_rec_ ~ 100 ms and the slow τ_rec_ ~ 1000 ms) extracted from the Y512A current recovery process (left panel of Figure 4D) are an order of magnitude larger than those of C212S. Both time constants from Y512A are not voltage dependent, while the fractions of the two current recovery components are, with the fraction of the slow component increasing while that of the fast component decreases with a more depolarizing V_m_ (right panel of Figure 4D). It appears that upon membrane depolarization, both C212S and Y512A mutants can enter deeper inactivated states (namely, deeper slow-gate closed states), therefore leading to a slower current recovery.

**Figure 5:**
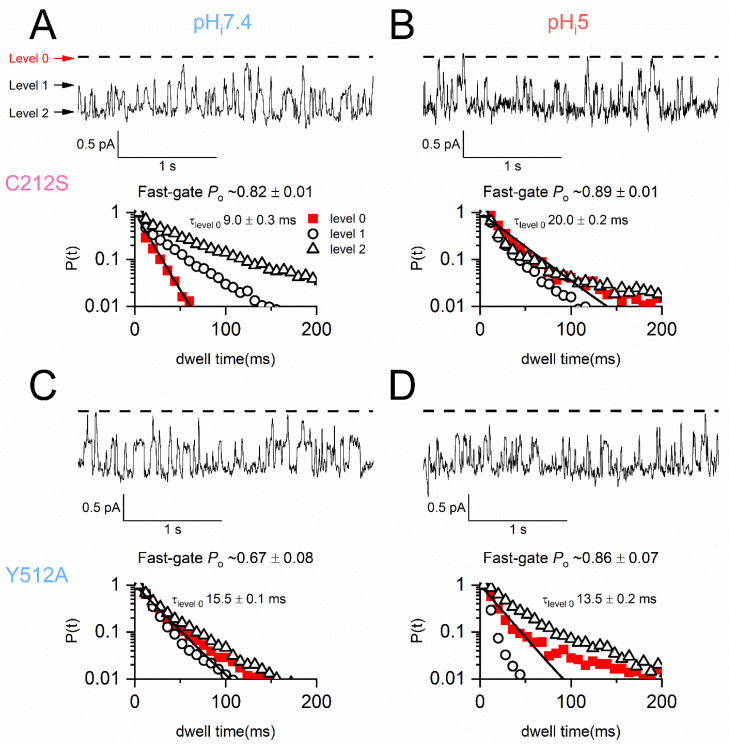
An analysis of fast gating from single-channel recording traces of C212S and Y512A. All experiments were conducted at V_m_ = −60 mV in symmetrical 140 mM NMDG-Cl. The dash lines indicate the zero-current level. (**A**,**B**) C212S recordings at pH_i_ 7.4 (**A**) and pH_i_ 5.0 (**B**). A total duration of 1146 s and 1043 s for pH_i_ 7.4 and 5.0, respectively. The cumulative dwell-time histograms for three current levels are shown only for events less than 200 ms. (**C**,**D**) Y512A recordings at pH_i_ 7.4 (**C**) and pH_i_ 5.0 (**D**). A total duration of 977 s and 899 s for pH_i_ 7.4 and 5.0, respectively. A cutoff at 200 ms in the x-axis is also used in the cumulative dwell-time histograms for the Y512A. Because the average duration of the fast-gate closed event is ~10 ms [19], the fast-gate *P*_o_ in all panels was calculated after excluding nonconducting events longer than 50 ms. In all four panels, mono-exponential fit of the nonconducting events (red solid squares) is plotted as a black line and the time constant of the exponential fit is given (τ_level 0_).

It has been reported that the slow-gate closure in the C212S and Y512A mutants is suppressed [35,37]. Therefore, it might be arguable that the current inhibition in these two mutants by H^+^_i_ does not result from a gating effect but could come from a reduction of the single-channel conductance. To differentiate a gating effect from a permeation effect, single-channel current recordings of the C212S and Y512A mutants were compared at pH_i_ 7.4 and 5.0. As shown in Figure 5A,C, the fast-gating activities reveal three current levels in the single-channel recording traces of C212S and Y512A. At pH_i_ = 7.4, nonconducting events longer than 100 ms are not detected in C212S (see the red squares in the dwell-time histogram in Figure 5A), an observation similar to the C212S behavior reported in the literature [35]. However, longer nonconducting events appear at pH_i_ = 5.0, as indicated by a dwell-time distribution of the nonconducting events deviating from a mono-exponential fit (Figure 5B). In Y512A, robust fast-gating activity is also observed, and the dwell-time histogram for nonconducting events less than 200 ms (see the red squares in Figure 5C) appears not to be a single–exponential distribution, indicating these nonconducting events consist of some slow-gate closed events. At pH_i_ = 5.0, the multi-exponential distribution of the nonconducting events becomes even more apparent, reflecting the presence of more slow-gate closed events. 

**Figure 6:**
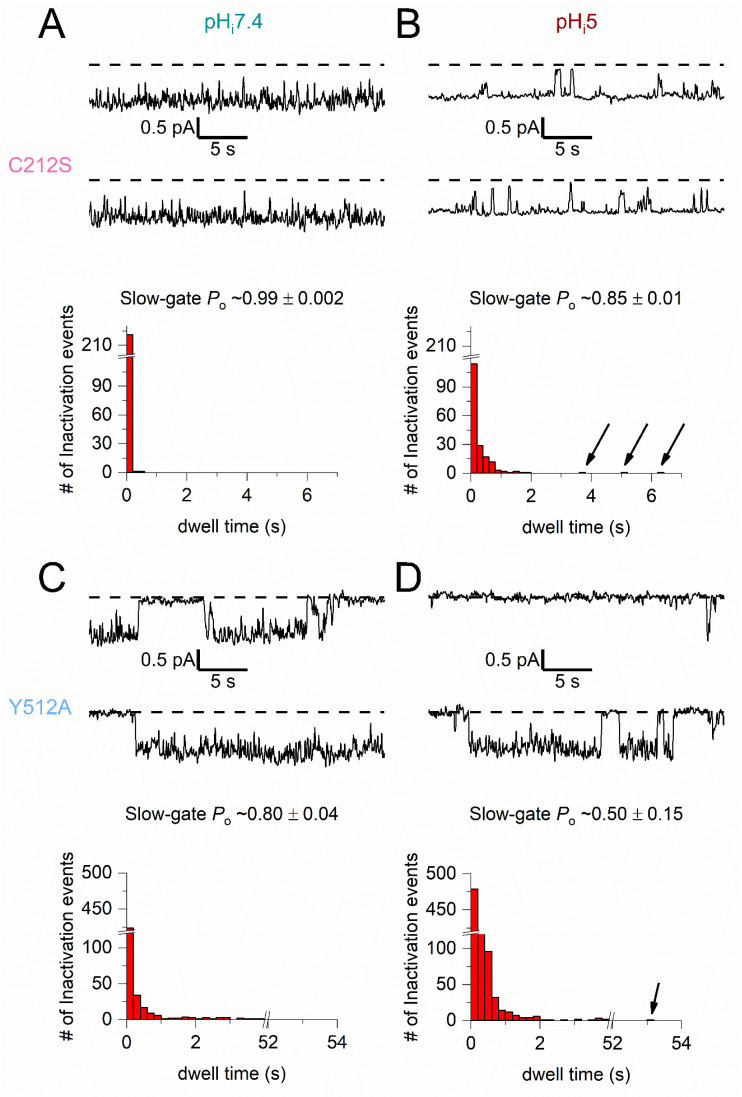
The slow gating of C212S and Y512A at pH_i_ 7.4 and 5.0 at the single-channel level. V_m_ = −60 mV. Recording traces were filtered at 5 Hz (see Section 4 for analysis procedures). (**A**,**B**) **Top**: The continuous one-minute single-channel recording traces of C212S at pH_i_ of 7.4 (**A**) and 5.0 (**B**). **Bottom**: The dwell-time histogram at pH_i_ 7.4 for 442 nonconducting events (from 1033 s of recording traces) and that at pH_i_ 5.0 for 368 nonconducting events (from 404 s of recording traces). The black arrows in the dwell-time histogram in B denote three nonconducting events with durations of 3.7, 5.1, and 6.3 s, respectively. (**C**,**D**) **Top**: The continuous one-minute single-channel recording traces of Y512A at pH_i_ of 7.4 (**C**) and 5.0 (**D**). Bottom: The dwell-time histograms for 1045 nonconducting events at pH_i_ 7.4 (from 863 s of recording traces) versus 1747 nonconducting events (from 888 s of recording traces) at pH_i_ 5.0. Notice that an axis break is introduced in the horizontal axis of the dwell-time histograms in (**C**,**D**) to show the long-lived inactivation events. For example, the arrow in the dwell-time histogram in (**D**) denotes a ~53.1 s nonconducting event. A break is also introduced in the y-axis of all of the histograms.

To directly observe the slow-gate closed events in C212S and Y512A, we digitally filtered the single-channel recording trace at 5 Hz (Figure 6). Such heavy filtering generates recording traces with only two current levels, representing the closed or open state of the slow gate. Compared to the recording at pH_i_ = 7.4 (Figure 6A), the recording trace of C212S at pH_i_ = 5.0 (Figure 6B) shows more nonconducting events of a longer duration (compare dwell-time histograms between Figure 6A,B). These nonconducting events, albeit short (mostly ~200–1000 ms), are much longer than the fast-gate closed events [35], suggesting that they are the inactivation events. The slow gating behavior of Y512A is illustrated in Figure 6C,D. At pH_i_ = 7.4, Y512A already exhibits long nonconducting inactivation events (Figure 6C), and the duration of these nonconducting events are much longer than those observed in the C212S mutant at the same pH_i_. The inactivation of Y512A is more pronounced at pH_i_ 5.0 (Figure 6D). Note that the single-channel conductance of C212S and Y512A is not altered by an acidic pH_i_ (Figure 6B,D), confirming the notion that the effect of H^+^_i_ is facilitating the closing of the slow gate but not reducing the channel conductance.

### 2.3. Analyzing Recovery from H^+^_i_ Inhibition in Mutants Missing E_gate_

**Figure 7:**
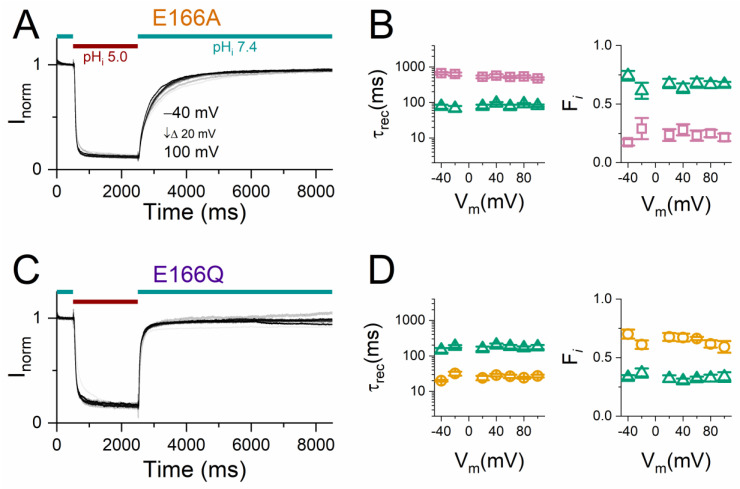
The current recovery kinetics of the E_gate_-removed mutants of CLC-0. (**A**,**C**) A time course of the acid-induced inhibition and current recovery of E166A (**A**) and E166Q (**C**). The same voltage protocols as those used in Figure 4. The red and green horizontal lines represent the patch exposure to pH_i_ = 5.0 and pH_i_ = 7.4, respectively. For comparison, the current is normalized to the current immediately before the application of the pH_i_ 5 solution. Note that the normalization procedure turns negative currents obtained at V_m_ < 0 to the positive values presented. (**B**,**D**) The averaged τ_rec_ (**left panels**) and fractions (**right panels**) of the two exponential components from a bi-exponential fit of the current recovery process. The data in panels (**B**) (E166A) and (**D**) (E166Q) were from experiments similar to those shown in panels (**A**,**C**), respectively (n = 5–6). Open gold circles, open green triangles, and open purple squares represent the ~10 ms, ~100 ms, and ~1000 ms exponential components, respectively.

The structure of CLC proteins suggest that the conserved glutamate residue (E166 of CLC-0) acts as the fast gate of the pore. Therefore, the negatively charged sidechain of this glutamate is called E_gate_ [21]. To examine whether H^+^_i_ can inhibit the E_gate_-removed mutants, we studied the H^+^_i_ inhibition of E166A and E166Q mutants in the same manner as described above for C212S and Y512A (Figure 7A,C). Surprisingly, the two E166 mutants were robustly inhibited by an acidic pH_i_. By fitting the current recovery in E166A, two recovery components comparable to those of Y512A were obtained, with τ_rec_s of ~100 ms and ~1000 ms (left panel of Figure 7B). The current recovery of E166Q appears to be faster than that of E166A and can be fit with a mono-exponential function. If a bi-exponential function is also used to fit the E166Q current recovery, two components like those of C212S (τ_rec_ ~10 and 100 ms) are obtained (left panel of Figure 7D). However, unlike C212S and Y512A, the fractions of both the fast and the slow recovery components in E166A and E166Q are voltage independent (right panels of Figure 7B,D).

We previously proposed a simple three-state model of inactivation [39], where the open channel is first inhibited by H^+^_i_ (inhibition) followed by a protein conformational change into a deeper inactivated state (inactivation):




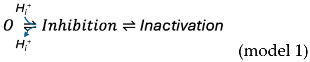




**Figure 8:**
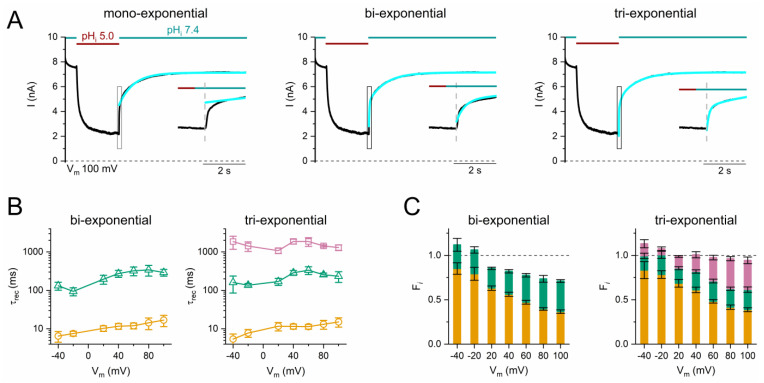
Comparison of various exponential curve fits of the current recovery kinetics of C212S, with a symmetrical 140 mM [Cl^−^] in all recordings. (**A**) A representative trace of the current inhibition and recovery of C212S obtained at +100 mV. The current recovery processes were fit with a mono- (**left panel**), bi- (**middle panel**), or tri-exponential (**right panel**) function. The black traces illustrate the original recording trace, while the blue traces represent the exponential fits. The curve fitting in the box plotted in the main panel is expanded in the insets. Notice that the beginning of current recovery is not fit well with a mono- or a bi-exponential function. (**B**) The average τ_rec_ from the bi- (**left**) and tri- (**right**) exponential curve fitting as a function of V_m_. Each exponential component is depicted with a symbol of a distinct color. (n = 5–6) (**C**) A summation of the fractions of all exponential components derived from bi- (**left panel**) and tri-exponential (**right panel**) curve fitting as a function of V_m_. The colors representing each exponential component are the same as in (**B**) (n = 5–6). Symbols and color schemes used in this Figure are the same as those in Figure 4 and Figure 7.

The two kinetic components of current recovery are consistent with two nonconducting states in model 1, namely, the “inhibited” and “inactivated” states. However, the time constants of two current recovery components vary in the four mutants studied—three roughly similar exponential time constants (~10 ms, ~100 ms, and ~1000 ms) were observed in the current recovery across the various mutants. This may suggest three instead of two nonconducting states. Careful examination of the fractions of the two recovery components shows that the summation of the two fractions in Y512A reaches unity at all V_m_s (right panel of Figure 4D), whereas the summation of those in C212S does not reach unity, especially with a V_m_ > +20 mV (right panel of Figure 4B). This indicates that a bi-exponential fit may not sufficiently describe the recovery process of C212S. To examine this possibility, the current recovery of C212S at +100 mV was compared among the fits with mono-, bi-, and tri-exponential equations (Figure 8A). With a tri-exponential fit, the two faster τ_rec_s from the three exponential fits are similar to those from the bi-exponential fit (Figure 8B, gold circles and green triangles), and the sum of the three recovery fractions is close to unity (right panel of Figure 8C). The additional slowest component in C212S (τ_rec_ ~1000 ms) appears to be comparable to the slow τ_rec_ observed in Y512A (left panel of Figure 4D, purple squares). The fraction of this slowest component with a τ_rec_ ~1000 ms in C212S becomes larger with a more positive V_m_, consistent with the idea that membrane depolarization drives the channel into deeper inactivated states. These results indeed suggest that the inactivation process of CLC-0 may include at least three nonconducting inactivated states: 
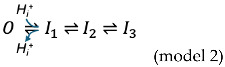


### 2.4. C212S Mutation Slows down the Transition from the I_1_ to I_2_ State

Although the C212S and Y512A mutations are both less sensitive to H^+^_i_ inhibition than the WT channel [39], their current recovery processes appear to be different—a ~10 ms recovery component is observed in the C212S mutant but not in Y512A. If the channel return from I_1_ to the conducting state (the “O” state in model 2) is fast (for example, τ ~10 ms) and if the transition from I_1_ to I_2_ is slow, the ~10-ms recovery component would become apparent as observed in the C212S mutant. To test this possibility, we varied the acidic pH_i_ exposure duration followed by assessing the current recovery process.

**Figure 9:**
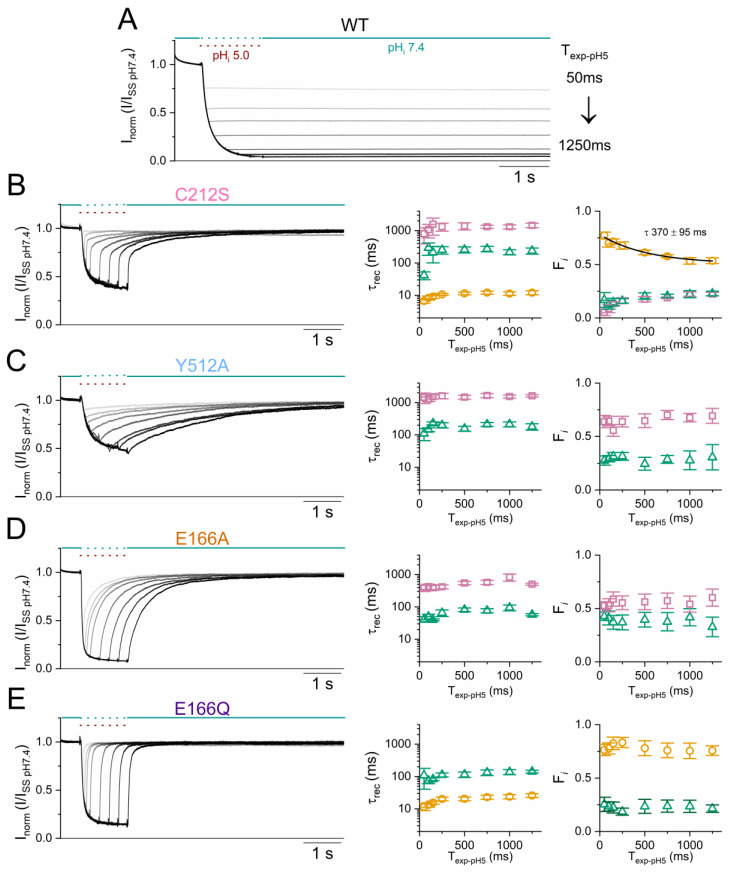
Assessing the kinetics of entering deep inactivated states in WT and mutant CLC-0 channels. All experiments were performed in symmetrical 140 mM [Cl^−^] at V_m_ = +40 mV. The current recovery kinetics of all mutants are fit with a bi-exponential function with the exception of C212S, which was fit with a tri-exponential function. (**A**) The WT CLC-0 current inhibited by acidic solution with various exposure durations. The two dotted colored lines indicate the variation of the time in exchanging solutions between pH_i_ 7.4 (sea-green) and pH_i_ 5.0 (wine). The exposure duration of the pH_i_ 5.0 solution ranges from 50 ms to 1250 ms. Note that the WT current does not recover after removing the acidic solution. (**B**–**E**) The **left panels** show the current inhibition and recovery from applying pH_i_ 5.0 solution with variable exposure duration as in (**A**). The **right panels** show the time-dependent τ_rec_ and fractions of the exponential components by fitting the current recovery process. The exponential decay of the amplitude of the fast exponential component (gold open circles) of C212S has a time constant of ~370 ms (solid black curve in the **right panel** of (**B**)). Note that the amplitudes of the fast exponential components in the other three mutants (**right panels** of (**C**–**E**)) are not time dependent (n = 5–6). Symbols and color schemes used in this Figure are the same as those in Figure 4, Figure 7 and Figure 8.

Regardless of the exposure duration, no current recovery was observed in WT CLC-0 upon removing the acidic pH_i_ (Figure 9A). In C212S, the fraction of the fast component decreases with a longer exposure to acidic pH_i_, and a mono-exponential fit of this decrease of the fast recovery fraction shows a time constant of ~370 ms (right panel of Figure 9B). In Y512A and the two E166 mutants, varying the acidic pH_i_ exposure duration yields no difference in the fractions of the current recovery components (Figure 9C–E, right). These results suggest that the C212S mutation, but not the other three mutations, slows down the transition from the I_1_ to the I_2_ state. Therefore, if the C212S mutant is only briefly exposed to the acidic pH_i_ (for example, <100 ms), most of the channels stay in the I_1_ state and can readily return to the open state with a τ_rec_ of ~10 ms. The lack of a recovery component with a τ_rec_ of 10 ms in Y512A suggests that the transition from the I_1_ to I_2_ state may be faster than the shortest acidic pH_i_ exposure duration (50 ms) in our experiments. The two τ_rec_s of E166A are similar to those of Y512A, lacking the ~10-ms component. However, the recovery process of E166Q seems to display mostly the ~10-ms recovery component. The reason for the difference in the τ_rec_ between E166A and E166Q is unknown. Nonetheless, because both E66A and E166Q are robustly inhibited by H^+^_i_, it appears that an intact E_gate_ is not required for the channel inactivation if the H^+^_i_ inhibition of CLC-0 is indeed due to the closure of the slow gate.

### 2.5. Increasing [Cl^−^] also Favors the Slow-Gate Opening in CLC-0 Mutants

**Figure 10:**
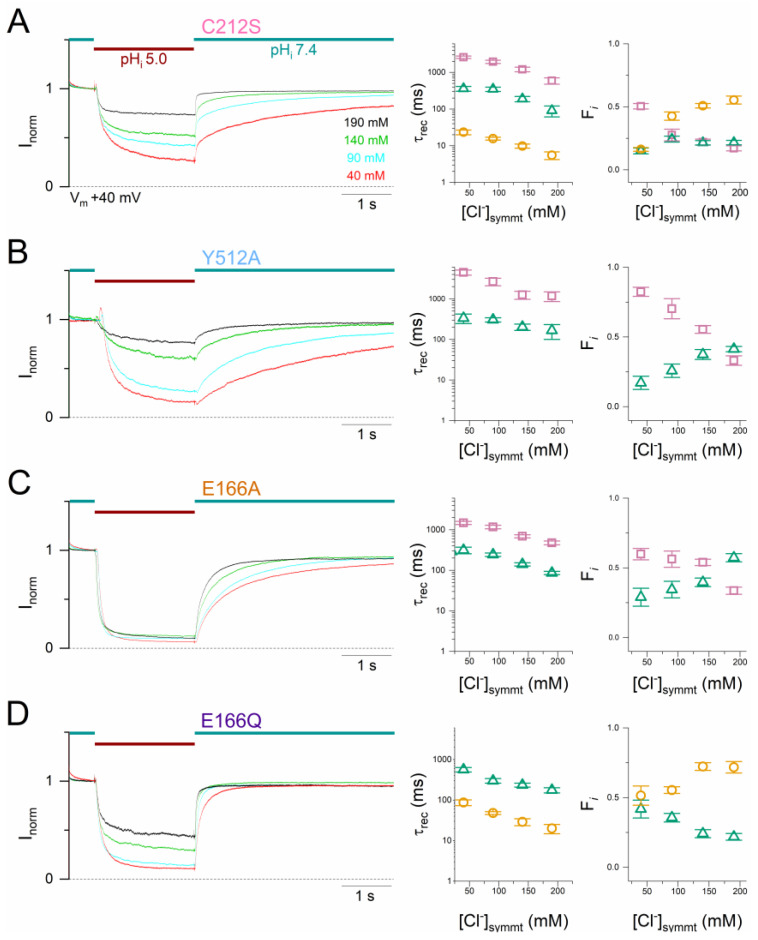
The current recovery from the acid-induced inhibition in mutant channels depends on [Cl^−^]. All experiments were conducted at V_m_ = +40 mV in symmetrical [Cl^−^]. The current recovery kinetics of C212S (A) were fit with a tri-exponential function, while those of Y512A (B), E166A (C), and E166Q (D) were fit with a bi-exponential function. The time course of the current inhibition and recovery under various symmetric [Cl^−^] (in mM): 40 (red), 90 (cyan), 140 (green), and 190 (black) are illustrated in the left panels, while the averaged τ_rec_ and the fraction of each exponential components from fitting the current recovery process are presented in the middle and right panels, respectively (n = 3–10). Symbols and color schemes used in this Figure are the same as those in Figure 4, Figure 7, Figure 8 and Figure 9.

**Figure 11:**
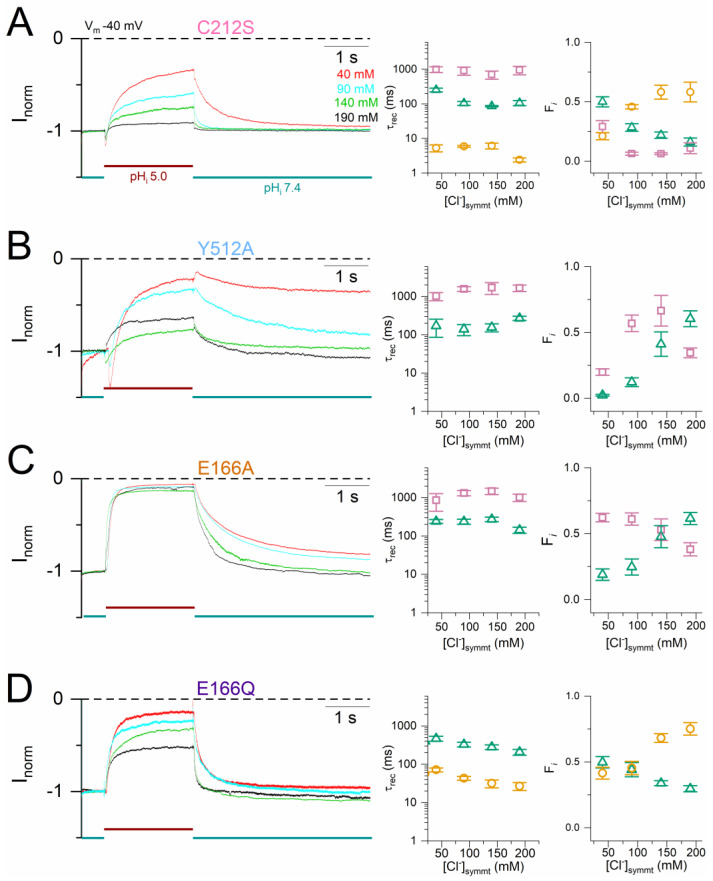
The current recovery from the acid-induced inhibition in mutant channels depends on [Cl^−^]. The data were obtained at V_m_ = −40 mV in symmetrical [Cl^−^]. Similar to the analysis in Figure 10, the current recovery kinetics of C212S (**A**) were fit with a tri-exponential function, and those of Y512A (**B**), E166A (**C**), and E166Q (**D**) were fit with a bi-exponential function (n = 3–7). Symbols and color schemes used in this Figure are the same as those in Figure 4, Figure 7, Figure 8, Figure 9 and Figure 10.

It has been shown that the slow-gate closure (namely channel inactivation) of WT CLC-0 is favored when [Cl^−^] is reduced and/or when the V_m_ is depolarized [19,32,34]. The recovery of WT CLC-0 from H^+^_i_ inhibition is also slower with reduced [Cl^−^] (Figure 2A), and with a more depolarized V_m_ (Figure 1C). To study the interaction between [Cl^−^] and V_m_, the kinetics of mutant channels were examined in various symmetrical [Cl^−^] at +40 mV (Figure 10) and at −40 mV (Figure 11), and the current recovery was fit with bi- or tri-exponential functions. The overall recovery process of these mutants becomes faster in higher [Cl^−^] similar to that observed in WT CLC-0 (Figure 2A), as indicated by a slightly shortened τ_rec_ and an increase of the fraction of the fastest recovery component (the ~10 ms component in C212S and E166Q and the ~100 ms component in Y512A and E166A). Meanwhile, the fraction of the slow recovery components (the ~100 ms component in E166Q and the ~1000 ms component in C212S, Y512A, and E166A) decreases proportionally with increasing [Cl^−^]. These results indicate that increasing [Cl^−^] also increases the rate of current recovery from acidic pH_i_ inhibition in these mutants.

## 3. Discussion

### 3.1. Membrane Hyperpolarization and Outward Anion Flux Favor CLC-0’s Slow-Gate Opening

In Kwon et al. [39], it was found that closing the slow gate of CLC-0 by H^+^_i_ is not voltage dependent, while recovery from H^+^_i_ inhibition (which represents the opening of the slow gate) depends significantly on V_m_. Experiments in the present study further show that the recovery from H^+^_i_ inhibition in WT CLC-0 is facilitated by a higher [Cl^−^] (Figure 2A). One explanation for the effects of V_m_ and [Cl^−^] on the current recovery rate is that the Cl^−^ flux through the channel pore is the ultimate critical factor. This speculation results from the observation that current recovery rates were significantly different at a constant V_m_ in various [Cl^−^] conditions. It appears that the ionic condition creating a greater outward Cl^−^ flux accelerates the rate of current recovery (Figure 2), and other permeant anions, like SCN^−^ and Br^−^, also exert similar effects (Figure 3). The dependence of the current recovery rate on the Cl^−^ flux through the pore is also observed in the mutant channels (Figure 10 and Figure 11). 

The possibility that an outward anion flux opens the gate of CLC channels has been proposed in CLC-2 by others [40,41]. It has long been observed that the opening of CLC-2 at a neutral pH_i_ is only possible with membrane hyperpolarization. Although the kinetics of the CLC-2 gating appear to be faster than those of the slow gating of CLC-0 in a neutral pH_i_, CLC-0’s slow gating can be fast in an acidic pH_i_ [39], raising a possibility that the slow-gating mechanism of CLC-0 may be similar to the hyperpolarization-activated gating mechanism of CLC-2. Interestingly, the CLC-2 mutations corresponding to the Y512 and E166 mutations of CLC-0 have also been shown to alter CLC-2 channel gating [41]. De Jesus-Perez et al. suggested that outward permeant anions open the gate of CLC-2 by electrostatic and steric interactions [41]. Similarly, the recovery of the CLC-0 current from acidic pH_i_ inhibition (the slow-gate opening) could also result from the electrostatic repulsion of the permeant anions with the E_gate_. 

### 3.2. Current Recovery Processes in CLC-0 Mutants Support Multiple Inactivated States

Because the current recovery from H^+^_i_ inhibition in WT CLC-0 does not occur at a positive V_m_, the mutant channels provide an opportunity to examine the current recovery process at a positive V_m_. One immediate insight from the previous study [39] was that the inhibition of CLC-0 by H^+^_i_ requires at least two steps: the channel is first inhibited by H^+^_i_, followed by a channel inactivation step (model 1). Concluding the presence of two nonconducting states was based on the observation that the recovery of the mutant channels at a positive V_m_ cannot be described well by a mono-exponential function. By analyzing the current recovery processes of various mutant channels in the present study, three temporally distinct components can be identified with τ_rec_s of ~10, ~100, and ~1000 ms, respectively (Figure 4 and Figure 7), indicating that at least three inactivated states (I_1_, I_2_, and I_3_) are necessary (see model 2). These time constants might correspond to the time required for the channel to go from the three nonconducting states back to the open state. However, WT CLC-0 does not show significant current recovery at a positive V_m_, suggesting that the WT channel could enter even deeper inactivated states beyond I_3_. The four-state model (model 2), therefore, is likely just a simplified scenario in describing the slow-gating mechanism of CLC-0.

In C212S, all three current recovery components are present, while only two recovery components were observed in Y512A—those with τ_rec_s of ~100 ms and ~1000 ms. The τ_rec_s of all recovery components in C212S and Y512A do not significantly vary with V_m_. Rather, it is the fractions of the various components that change with the V_m_—membrane depolarization decreases the fraction of the fast component accompanied with increases of the slow components (Figure 4). It appears that membrane depolarization renders the channel access to deeper inactivated states. The fraction of the fastest recovery component (namely, that with a τ_rec_ of ~10 ms, which likely corresponds to the transition from the I_1_ to the O state) also decreases with an increase of the acidic pH_i_ exposure time in C212S, suggesting that the number of C212S channels in the I_1_ state decreases with longer acidic pH_i_ exposure. However, no recovery exponential component with a τ_rec_ of ~10 ms is observed in Y512A (Figure 9C), indicating that a transition from I_1_ to I_2_ in Y512A (and also in WT CLC-0) must be less than 50 ms, the shortest exposure duration of acidic pH_i_ in these experiments. A time constant of the exponential decay of ~370 ms in the C212S fastest recovery component indicates that the transition from I_1_ to I_2_ in C212S is hampered. Thus, although both C212S and Y512A mutations affect the slow gating of CLC-0, they appear to hamper the slow-gating transition through different mechanisms. C212S appears to affect the transition from I_1_ to I_2_, while Y512A mutation seems to slow down the transition from I_3_ to even deeper inactivated states (not represented in model 2). Additionally, neither the thiol group of C212 nor the phenol group of Y512 serves as the protonation site for initiating the inactivation process because mutations of these two residues do not eliminate the acidic pH_i_ inhibition of the channel current.

### 3.3. Removal of the E_gate_ Charge Does Not Eliminate CLC-0’s Slow Gating

The movement of the sidechain of E166 (E_gate_) in CLC-0 has been thought to be responsible for the fast-gating mechanism. Introducing a neutrally charged residue to this position results in a constantly open channel [24,52]. Because the sidechain of the conserved glutamate residue appears to be the only obstruction of ion flow through the pore, it raises the question of whether removing the negative charge of E_gate_ affects the slow gating. As shown in Figure 7A,C, both E166A and E166Q display significant acidic pH_i_ inhibition even greater than those in C212S and Y512A. Although a mono-exponential function might sufficiently describe the recovery process in these two E166 mutations, a bi-exponential fit of the current recovery processes was conducted, similar to the analysis of Y512A. The curve fitting reveals two recovery components with τ_rec_s of ~10 ms and ~100 ms in E166Q and τ_rec_s of ~100 ms and ~1000 ms in E166A. Both the τ_rec_ and the fraction of the current recovery components are independent of the V_m_ (Figure 7B,D). These results indicate that the E_gate_ is not the protonation site for acidic pH_i_ inhibition, and removal of E_gate_ does not eliminate the transition to inactivated states. In these two mutants, the occlusion structure in the pore has been removed [21], and yet, the mutants are still inhibited by the acidic pH_i_, raising a question about the nature of the I_1_ state. Perhaps the nonconducting I_1_ state does not result from an occluded pore but rather from a blocked state of the pore from tighter anion binding in the pore promoted by the acidic pH_i_. The increased stability of the inactivated state of the channel in low [Cl^−^] conditions (Figure 10 and Figure 11), which likely would reduce the electrostatic interaction of Cl^−^ ions in the pore due to a smaller Cl^−^ flux, is consistent with the speculation that the I_1_ state may result from the block of the pore by permeant anions. If this is the case, the pore blockade by ion binding would likely be affected by the pore geometry as well as the hydrophobicity of the pore residues. It has been shown that the affinities of amphiphilic pore blockers in E166A and E166Q mutants, in which the sidechains of residue 166 (alanine vs. glutamine) have different lengths and hydrophobicity, differ by three orders of magnitude [52]. It is therefore not surprising that these two mutants reveal very different kinetics of current recovery from H^+^ inhibition.

### 3.4. Single-Channel Recordings Provide Support to the Macroscopic Current Behaviors

The inactivation-suppressed CLC-0 mutants, namely C212S and Y512A, are less sensitive to acidic pH_i_ inhibition than the WT channel [39]. The gating behaviors of the C212S mutant had been examined at the single-channel level, and it was speculated that the slow gate of C212S is constantly open because no apparent inactivation events were observed at a neutral pH_i_ [35]. The Y512A mutant was also shown to be more difficult to enter the inactivated state based on its low sensitivity to extracellular zinc inhibition [34,37]. However, the Y512A mutant still shows frequent long inactivation events at pH_i_ 7.4 (Figure 6C). Because C212S is inhibited by acidic pH_i_, it is reasonable to argue that the slow-gate closure does occur in C212S even at a neutral pH_i_, although short inactivation events of tens of ms duration (corresponding to the channels residing at the I_1_ state in model 2) may not be readily distinguished from the fast-gate closing events. Comparing the dwell-time histograms of C212S in Figure 6A,B shows that the nonconducting events longer than 200 ms become apparent at pH_i_ = 5. Similarly, the acidic pH_i_ also significantly increases the number of nonconducting events longer than 200 ms in Y512A (Figure 6C,D). Here, the use of 200 ms as a cutoff to distinguish short (fast-gate closure) from long nonconducting inactivation events is arbitrary. In Y512A, it is much easier to distinguish the inactivation events from the fast-gate closed events as the former are much longer than the latter. In C212S, those nonconducting events less than 200 ms at pH_i_ = 7.4 ([H^+^] ~0.04 μM) may also include events of slow-gate closure. Notice that a comparison of the dwell-time histograms of the inactivation events between C212S and Y512A at pH_i_ = 7. 4 (Figure 6A versus Figure 6C) shows that the inactivation events of Y512A are significantly longer, consistent with the idea that the Y512A mutant channel tends to enter deeper inactivated states than the C212S mutant, leading to an overall slower recovery from the acidic pH_i_ inhibition observed in the macroscopic current recordings. 

In conclusion, we propose that the binding of H^+^_i_ to CLC-0 increases the affinity of the channel pore for the permeant anions. A longer resident time of permeant anions in the pore leads to a blocked state (I_1_). Subsequently, the blocked channel can transition into deeper inactivated states (I_2_, I_3_, and possibly deeper inactivated states) through a voltage-dependent process. Despite extensive research on CLC-0 over the years, the exact mechanism of slow gating remains elusive. The present study provides insights that multiple inactivated states exist and transitions among these states are affected by anion permeation and H^+^_i_ modulation. However, important questions remain to be answered. For example, the protonation site(s) responsible for H^+^_i_ inhibition as well as the structural characterization of the multiple inactivated states have yet to be determined. Future experiments, guided by the insights obtained from this study, will contribute to establishing a more comprehensive understanding of the slow gating of CLC-0.

## 4. Materials and Methods

### 4.1. Molecular Biology and Channel Expression

All experiments in this study were conducted using channels expressed in human embryonic kidney 293 (HEK293) cells (for macroscopic current recordings) or in *Xenopus* oocytes (for single-channel recordings). For macroscopic electrophysiological recordings, cDNA constructs of WT and mutant CLC-0 channels were subcloned in the pIRES2-EGFP vector (Clontech/Takara Bio, Mountain View, CA, USA) containing internal ribosome entry sites (IRES) and an enhanced green fluorescent protein (EGFP) gene. The mutations of CLC-0 were made using the QuikChange site-directed mutagenesis kit (Agilent Technologies, Santa Clara, CA, USA), and the mutated nucleotide sequences were verified via a commercially available sequencing service. For channel expression, the cDNA was transiently transfected to HEK293 cells using Lipofectamine 3000 kit (Invitrogen, ThermoFisher Scientific, Waltham, MA, USA) per manufacturer’s protocols. The HEK293 cells were grown in Dulbecco’s Modified Eagle’s Medium (DMEM) containing high glucose and supplemented with 10% fetal bovine serum and 1% streptomycin/penicillin (Gibco, ThermoFisher Scientific, Waltham, MA, USA). The cells were incubated in a 37 °C incubator with 5% CO_2_. Following transfection, the cells were seeded onto the coverslip pre-treated with poly-L-lysine and were further incubated at 37 °C until the experiments were performed 1–2 days later. 

For single-channel recording experiments, the cDNAs of C212S and Y512A mutants were constructed in the pBluescript vector, and the capped mRNAs were synthesized using commercially available mMessage mMachine T3 Transcription kit (Invitrogen, ThermoFisher Scientific, Waltham, MA, USA). Fifty μL of mRNA was injected into *Xenopus* oocytes using a nanoliter injector (World Precision Instrument, Inc., Sarasota, FL, USA). To obtain an optimal channel density suitable for single-channel experiments, the RNA was diluted according to the whole oocyte current recorded using the OC-725C two-electrode voltage clamp amplifier (Warner Instrument/Harvard Apparatus, Hamden, CT, USA). The injected oocytes were incubated in ND-96 at 18 °C for two to three days. Oocytes expressing 4–8 μA whole-cell current were used for single-channel recordings.

### 4.2. Electrophysiological Recordings

**Macroscopic current recordings:** Immediately prior to the experiment, the coverslips with transfected HEK293 cells were moved to the recording chamber containing various recording solutions (see below) on a Leica DM IRB inverted microscope equipped with a GFP filter (Chroma Technology, Bellows Falls, VT, USA). Transfected cells were identified based on the green fluorescence on the cells excited by the XT640-W LED light (Lumen Dynamics, Mississauga, ON, Canada). Recording pipettes were fabricated from borosilicate glass capillaries (World Precision Instrument, Sarasota, FL, USA) using the PP830 electrode puller (Narishige, Amityville, NY, USA). When filled with recording solutions, the pipette resistance was 1.5–2.5 MΩ. Unless indicated elsewhere, the pipette solution contained 130 mM NaCl, 5 mM MgCl_2_, and 10 mM of HEPES (4-(2-hydroxyethyl)-1-piperazineethanesulfonic acid) buffered at pH 7.4.

All macroscopic recordings were conducted in excised inside-out membrane patches from HEK293 cells. The recording-chamber solutions, which are intracellular solutions, contained 130 mM NaCl, 5 mM MgCl_2_, 10 mM of pH buffers, and 1 mM EGTA (ethylene glycol-bis aminoethyl ether-tetraacetic acid). HEPES and MES (2-(N-morpholino)ethanesulfonic acid) were used to buffer the pH at 7.4 and at 5, respectively. In low chloride conditions, the osmolarity of the solutions is balanced by replacing NaCl with equimolar D-mannitol. The change in osmolarity shows no effect on the kinetics of current recovery from acid-induced inhibition (Figure S1, Supplemental Materials). All chemicals (American Chemical Society grade) were obtained from Sigma-Aldrich (St. Louis, MO, USA), VWR Chemicals (Radnor, PA, USA), or ThermoFisher Scientific (Waltham, MA, USA). The fast exchange of intracellular solutions was achieved using SF-77B solution exchanger (Warner Instrument/Harvard apparatus, Hamden, CT, USA). The Axopatch 200B voltage-clamp amplifier was used for recording, and the recorded current filtered at 2 or 5 kHz and was digitized at 5 kHz or 10 kHz using Digidata 1440A digitizing board controlled by pClamp 11 software (Axon Instruments/Molecular Devices, San Jose, CA, USA). 

**Single-channel recording experiments:** In preparation for single-channel recordings, *Xenopus* oocytes were transferred to a hypertonic solution (300 mM NaCl), and the vitelline membrane of oocytes was removed manually. Inside-out membrane patches were excised directly into the bath solution containing 130 mM NMDG-Cl (N-methyl-d-glucamine chloride), 5 mM MgCl_2_, 1 mM EGTA, and 10 mM HEPES (pH 7.4) or MES (pH 5). The pipette solution is the same as the bath solution at pH 7.4. Axopatch 200B and Digidata 1440A controlled by pClamp11 were used to conduct single-channel recordings. Upon successfully obtaining a single-channel patch, a constant clamped voltage of −60 mV was applied. The recorded current was first filtered by the built-in 1-kHz analog filter of the Axopatch 200B amplifier, followed by an additional 0.2 kHz digital filtering by the pClamp 11 software. The sampling rate was 2 kHz. 

### 4.3. Data Analyses

**Macroscopic current recordings:** To study the slow gating of CLC-0, an experimental protocol similar to that in the previous paper was used [39]. Briefly, before starting any experiment, negative voltage pulses (−100 mV for 50 ms) were applied multiple times to open the slow gate until the recorded current no longer increased. The slow-gate suppressed mutants, such as C212S [35], Y512A [37], and E166 mutants [24], did not require this current-activation procedure. The current was inhibited by applying a solution of acidic pH to the intracellular side of the membrane, and the current recovery process was examined after the intracellular solution was switched back to the one with neutral pH. Using Clampfit 11 software (Molecular Devices), the recovery kinetics were studied by performing exponential fit using up to three exponential terms (i = 1–3):

(1) $${I\left(t\right)}_{rec}=\sum_{i=1}^{n}A_{i}*e^{\frac{-t}{\tau_{i}}}+C,$$

where n is the number of exponential components, τ*_i_* and A*_i_* are the time constant and the amplitude of exponential component i of the recovery kinetics, and C is the offset. To compare the results from different sets of experiments, the fraction of component i (F*_i_*) was defined as the amplitude of component i normalized to the sum of the amplitudes of all exponential components according to Equation (2):

(2) $$F_{i}=\frac{A_{i}}{\sum_{i=1}^{n}A_{i}}.$$

**Single-channel recordings:** To analyze single-channel recording traces, digital filtering at 100 Hz or at 5 Hz was further applied to analyze the fast gating and the slow gating of the channel, respectively. The fast-gating activity exhibits three equidistant current levels, level 0, 1, and 2, corresponding to the opening of 0, 1, and 2 protopores. The fast-gating open probability (*P*_o_) was calculated based on the following equation:

(3) $$P_{o}=\frac{f_{1}}{2}+f_{2}$$

where *f*_1_ and *f*_2_ are the time fractions of level 1 and level 2, respectively. Nonconducting events longer than 50 ms were excluded before calculating the fast-gate *P*_o_. Dwell-time histograms of the events at the three current levels were compiled with a bin width of 8 ms (Figure 5). The dwell-time histogram of nonconducting events (namely, events at level 0) was fit with mono-exponential decay, which appears as a straight black line in the semi-log plot of the dwell-time histograms in Figure 5. 

For the analysis of the slow gating, the heavy filtering at 5 Hz eliminates short nonconducting events (which mostly are fast-gate closing events), and the three current levels in the recording trace collapses into only two current levels, namely, current levels corresponding to the closed and open states of the slow gate [35]. The time fraction of the open current level out of the total duration of the recorded traces was defined as the *P*_o_ of the slow gate. The dwell times of the nonconducting events were compiled with a bin width of 200 ms, and the number instead of the fraction of the nonconducting events is plotted in Figure 6. All data are presented as mean ± SEM. 

### 4.4. Homology Modeling of the CLC-0 Structure and Amino Acid Sequence Alignment

Two predicted structures of the *Torpedo* CLC-0 monomer (from *T. californica* and *T. marmorata*) exist in the AlphaFold website (https://alphafold.ebi.ac.uk) accessed on 19 October 2022. The CLC-0 structure of *T. californica* [53,54] was chosen to pair with the monomer structure of hCLC-1 to construct a hCLC-1/CLC-0 heterodimer shown in Figure 1A. The hCLC-1 structure (PDB code: 6QVC [16]) was obtained from the protein data bank (https://rcsb.org) accessed on 27 June 2022 and was used as a reference in the heterodimer construction. The structures of hCLC-1 and CLC-0 structure were aligned using the “matchmaker” tool in the UCSF Chimera program. Aligning pair of chains was iterated using the Needleman–Wunsch algorithm until the highest secondary structure score was reached. For the sequence alignment of CLC-0 and hCLC-1 (shown at the bottom of Figure 1A), the amino acid sequences of hCLC-1 and CLC-0 were aligned using the scoring function of ClustalWS in the Jalview program (https://jalview.org) accessed on 27 June 2022.

## Data Availability

The original contributions presented in the study are included in the article/Supplementary Materials; further inquiries can be directed to the corresponding author.

## Supplementary Materials

The supporting information can be downloaded at: https://www.mdpi.com/article/10.3390/ijms25147796/s1.

## Abbreviations

H^+^	protons
Cl^−^	chloride ions
Br^−^	bromide ions
SCN^−^	thiocyanate ions
H^+^_i_	intracellular protons
[H^+^]_i_	concentration of intracellular protons
[Cl^−^]_i_	concentration of intracellular chloride ions
[Cl^−^]_o_	concentration of extracellular chloride ions
pH_i_	intracellular pH
HEK	human embryonic kidney
C212S	C212S mutant of the *Torpedo* CLC-0 Cl^−^ channel
Y512A	Y512A mutant of the *Torpedo* CLC-0 Cl^−^ channel
E166A	E166A mutant of the *Torpedo* CLC-0 Cl^−^ channel
E166Q	E166Q mutant of the *Torpedo* CLC-0 Cl^−^ channel
hCLC-1	human CLC-1 chloride channel
WT	wild type
E_gate_	glutamate gate
I_norm_	normalized current
mV	millivolt
ms	millisecond
s	second
Hz	Hertz (1/sec)
V_m_	membrane voltage
*P*_o_	open probability
τ_rec_	time constant of the current recovery
F*_i_*	fraction of exponential component i
*f*_i_	time fraction of the current level i (i = 0, 1 or 2)
P(t)	Probability of events longer than time t
NMDG	N-methyl D-glucamine
EGTA	*ethylene*-bis(oxyethylenenitrilo)*tetraacetic acid*
HEPES	4-(2-hydroxyethyl)-1-piperazineethanesulfonic acid
MES	2-(N-morpholino)ethanesulfonic acid
