# Supplementary material for: Insights into CLC-0’s Slow-Gating from Intracellular Proton Inhibition
Source: Int J Mol Sci. 2024 Jul 16;25(14):7796. doi: 10.3390/ijms25147796 (PMC11276645; doi:10.3390/ijms25147796)
Supplement: Supplementary file 1 [file ijms-25-07796-s001.zip › ijms-3036976-supplementary.pdf]

Kwon H.C., Fairclough R.H., and Chen T.-Y.

Insights into CLC-0's slow-gating from intracellular proton inhibition

SUPPLEMENTAL MATERIALS

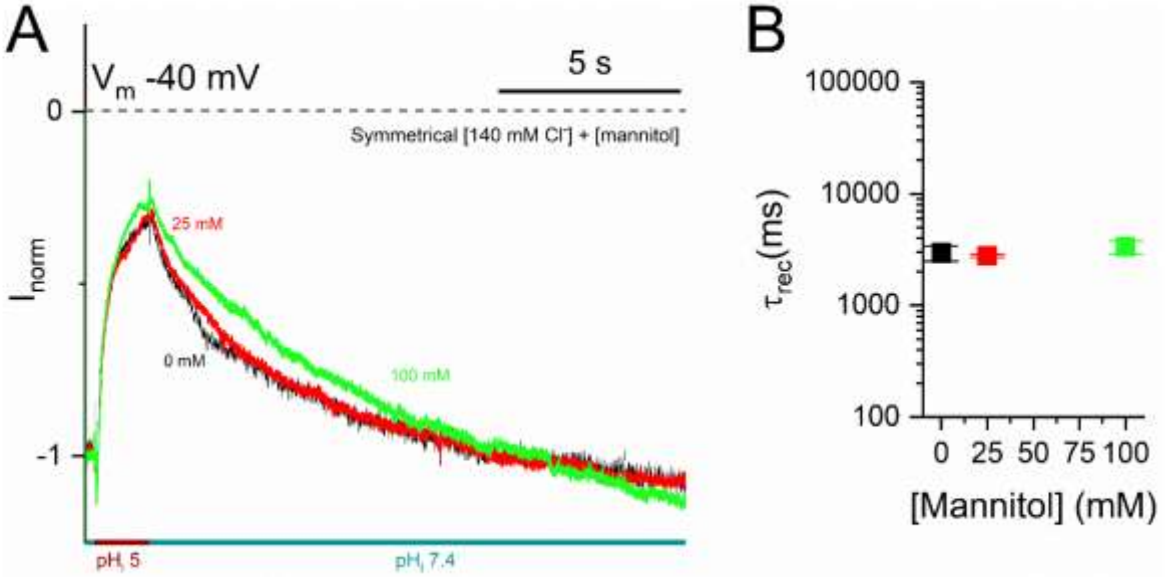

Figure S1. Little effect of osmolarity on the current recovery rate from acid-induced inhibition. **(A)** Acid-induce inhibition and recovery of WT CLC-0 at V_m = -40 mV and in symmetrical solutions containing 140mM symmetrical Cl^- and various [mannitol] (0 mM (black); 25mM (red); 100mM (green)). **(B)** τ_{rec} as a function of [mannitol].
